# Analysis of global burden of inflammatory bowel disease among adolescents and young adults from 1990 to 2021 and projections to 2040

**DOI:** 10.1186/s12889-025-24105-0

**Published:** 2025-09-24

**Authors:** Ziyu Yuan, Liji Chen, Chong Shan Ng, Peishan Zhuang, Chongyang Huang, Jun Wang, Yao He, Beiping Zhang, Cailing Zhong, Haiyan Zhang

**Affiliations:** 1 The Second Clinical Medical College, Guangzhou University of Traditional Chinese Medicine, Guangzhou, China; 2 Clinical College of Chinese Medicine, Hubei University of Chinese Medicine, Wuhan, China; 3 Department of Gastroenterology, The First Affiliated Hospital, Sun Yat- sen University, Guangzhou, China; 4 Department of Gastroenterology, The Second Affiliated Hospital of Guangzhou University of Traditional Chinese Medicine, Guangzhou, Guangdong Province 510120 China

**Keywords:** Inflammatory bowel disease, Adolescents and young adults, Global burden of disease, Epidemiology, Projection

## Abstract

**Background:**

Inflammatory bowel disease (IBD) is a chronic nonspecific intestinal disorder with peak onset ages in adolescents and young adults (AYA, aged 15–39) and a lifelong risk of recurrence. AYA constitute a significant and unique proportion of its disease burden, facing challenges in fertility preservation, psychosocial support, and economic impact. Accurate epidemiological insights are crucial for national disease control and prevention strategies, yet research on AYA IBD remains sparse. This study aims to assess the burden of AYA IBD from 1990 to 2021 by global, regional, national, socio-demographic index (SDI), sex and age group, and to predict the disease burden in 2040.

**Methods:**

Using data from the Global Burden of Disease (GBD) study, we conducted a comprehensive analysis of disability-adjusted life years (DALYs), deaths, incidence, and prevalence of AYA IBD from 1990 to 2021, with subgroup analyses by region, country, SDI, sex and age group. We calculated the estimated annual percentage changes (EAPCs) of age-standardized rates (ASRs) to assess global trends in AYA IBD burden from 1990 to 2021. We also examined the correlations between EAPC and ASR as well as SDI across countries and projected the disease burden to 2040 with Bayesian age-period-cohort (BAPC) model.

**Results:**

Globally, AYA IBD DALYs increased from 246,873.18 in 1990 to 320,607.21 in 2021. By 2040, DALYs are projected to continue rising steadily. East Asia saw the most marked increases in age-standardized incidence rate (ASIR) and age-standardized prevalence rate (ASPR), at 3.28% and 2.60%, respectively. Australia experienced the steepest rises in age-standardized DALYs rate (ASDR) and mortality rate (ASMR), at 1.19% and 2.33%, respectively. High SDI countries still had the highest ASDR (21.90/100,000), ASIR (13.65/100,000), and ASPR (109.31/100,000) in 2021. Middle SDI countries exhibited the largest ASDR, ASIR and ASPR increases. In 2021, the EAPCs of ASDR and ASMR were negatively correlated with SDI. Females had higher ASDR, ASMR and ASPR. The highest disease burden was observed in the 35–39 age group.

**Conclusion:**

AYA IBD represents a significant global public health burden, expected to persist through 2040. With the increasing aging population, policymakers should develop appropriate strategies to mitigate the burden of AYA IBD.

**Supplementary Information:**

The online version contains supplementary material available at 10.1186/s12889-025-24105-0.

## Background

Inflammatory bowel disease (IBD), encompassing Crohn’s disease (CD) and ulcerative colitis (UC), is a chronic nonspecific intestinal disorder with peak onset ages in adolescents and young adults and a lifelong risk of recurrence [1, 2]. Patients aged 15–39 years with IBD are defined as adolescents and young adults IBD (AYA IBD) [3]. Relative to disorders that primarily afflict the elderly, AYA IBD imposes greater demands in the following aspects: firstly, in the context of biologic therapy, fertility preservation should be considered, such as pre-pregnancy evaluation for females and guidance on sperm banking for males; secondly, long-term psychosocial support, like providing proper counseling to cope with academic or occupational stress; and thirdly, sustained management of the socioeconomic burdens, including higher medical costs and more severe work efficiency loss. These unique challenges underscore the pivotal role of addressing AYA IBD in advancing translational research and clinical care for IBD at large [1, 2, 4–8].

IBD is now recognized as a global health issue. Over the past few decades, IBD has been prevalent in developed countries, while its incidence has been gradually increasing in developing countries [9, 10]. However, there is a scarcity of comprehensive and systematic epidemiological analyses of AYA IBD across multiple dimensions, including the global, regional, national, sociodemographic index (SDI) levels sex and age group, as well as projections of future trends [10, 11]. Investigating the differences and trends in the disease burden of AYA IBD across these subgroups is critical, as it provides essential insights for informing future health planning strategies.

This study leverages the latest data from the Global Burden of Disease(GBD)database to comprehensively analyze the annual number of deaths, prevalence, incidence, and disability-adjusted life years (DALYs) of AYA IBD from 1990 to 2021, globally, with subgroup analyses by region, country, SDI, sex and age group, as well as their respective age-standardized rates (ASRs) and estimated annual percentage changes (EAPCs). We also analyzed the correlations between EAPC and ASR as well as SDI in 2021 and projected the global DALYs, deaths, incidence, and prevalence of AYA IBD, as well as their ASRs, from 2022 to 2040. Our study aims to fully investigate and analyze the disease burden of AYA IBD and provide projections for future trends to reduce the global burden of AYA IBD.

## Methods

### Data source

The data used in this study were obtained from the Global Health Data Exchange query tool (GHDx http://ghdx.healthdata.org/gbd-results-tool). The GBD study divides the world into 21 regions based on geographical location and epidemiological similarity. These regions are further described as 204 countries and territories. The IBD burden in each region and country allows us to identify the regions and countries with the highest disease burden and potential intervention targets. The 2021 GBD study was used to analyze the global, regional, and national IBD burden from 1990 to 2021. We obtained data on prevalence, incidence, DALYs and mortality annual values and rates with 95% uncertainty intervals (UIs) from the 2021 GBD, specifically for region, nation, SDI, age, and gender. Our analysis included IBD patients aged 15–39 and divided them into five age groups (15–19, 20–24, 25–29, 30–34, 35–39). In addition, the SDI is a composite metric developed by the Institute for Health Metrics and Evaluation to characterize regional development across social, demographic, and economic dimensions. It integrates three core components: per capita income, average educational attainment, and total fertility rate, weighted to reflect their interdependence in shaping societal progress. SDI ranges from 0 to 1, with values categorized into five tiers: low (e.g., Afghanistan), low-middle (e.g., India), middle (e.g., Brazil), high-middle (e.g., Ukraine), and high (e.g., Australia), to enable standardized cross-national comparisons in health and development research [10].

### Statistical analysis

ASRs and EAPCs were used to quantify the trends in AYA IBD burden. Given that the included data came from multiple populations across different age groups and changed over time, it was necessary to standardize and analyze the data to avoid errors. ASRs were used as the main indicator to estimate disease burden, calculated as follows: ASR = ΣiA a.i. wi/ΣiA wi × 100,000, where i represents the ith age group, a.i. and wi represent the age-specific rate and the world standard population of each age group, respectively. EAPC is widely used to summarize ASR trends over a certain period. A regression line was fitted to the natural logarithm of the rate, y = α + βx + e, where y = ln (ASR), x = calendar year, and e is the error term. The EAPC formula is 100 × [exp(β) − 1], with its 95% confidence intervals (CIs) derived from the linear regression model. If the 95% CI of EAPC is > 0, the indicator is increasing; if the 95% CI < 0, it is decreasing; if the 95% CI is zero, it is stable [12]. The correlations between EAPC and ASR as well as SDI in 2021 were analyzed and determined. To predict the future disease burden of AYA IBD, we applied the Bayesian age-period-cohort (BAPC) model. We conducted a BAPC analysis with integrated nested Laplace approximation (INLA). To ensure smoothing, BAPC models assume independent mean-zero normal distributions on the second differences of all effects [13, 14]. Specifically, the BAPC model assumes prior distribution of the age effect as follows:

$$\:f\left(\alpha\:|{k}_{\alpha\:}\right)\propto\:{k}_{\alpha\:}^{\frac{t-2}{2}}\text{exp}\left\{-\frac{{k}_{\alpha\:}}{2}\sum\:_{i=3}^{I}\:\:{\left[\left({\alpha\:}_{i}-{\alpha\:}_{i-1}\right)-\left({\alpha\:}_{i-1}-{\alpha\:}_{i-2}\right)\right]}^{2}\right\}$$

Considering that we are interested in the incident case counts for age group *α*, with a *t* period into the future, the following equation can be applied:

$$\:\text{l}\text{o}\text{g}\left({Y}_{a,p+t}\right)=\mu\:+{\alpha\:}_{a}+{\beta\:}_{p+t}+{\gamma\:}_{c+t}+{\delta\:}_{a,p+t},$$

Here, we add an independent random effect $\:{\delta\:}_{a,p+t}\sim\:N(0,{k}_{\delta\:}^{-1})$to adjust for overdispersion. Considering the smoothing assumption, the BAPC models assume prior distribution of the period effect as follows:

$$\:{\beta\:}_{p+t}|{\beta\:}_{1},\dots\:,{\beta\:}_{p},{k}_{\beta\:}\sim\:N\left((1+t){\beta\:}_{p}-t{\beta\:}_{p-1},{k}_{\beta\:}^{-1}(1+{2}^{2}+\cdots\:+{t}^{2})\right)$$

In this study, all data analyses were performed using R software package (version 4.2.3) and JD_GBDR (V2.22, Jingding Medical Technology Co., Ltd.) for graphical representation.

## Results

### Global burden and its trend of AYA IBD from 1990 to 2021, and projections to 2040

In 2021, the global burden of AYA IBD increased from 246,873.18 DALYs (95% UI, 201,290.63 to 296,020.06) in 1990 to 320,607.21 DALYs (95% UI: 257,687.65 to 396,103.28), representing a 29.9% increase. The age-standardized DALYs rate (ASDR) decreased from 11.26 per 100,000 (95% UI: 9.18 to 13.51) in 1990 to 10.78 per 100,000 (95% UI: 8.66 to 13.32) in 2021. From 1990 to 2021, the ASDR of AYA IBD decreased by an average of −0.20% annually (95% CI: −0.27 to −0.13). (Table 1).

**Table 1:** Summary of IBD in patients aged 15–39 Disability-adjusted life years and age-standardized disability-adjusted life years rates in 1990 and 2021

	1990 DALYs (95% UI)	2021 DALYs (95% UI)	1990 ASDR, per100,000 people (95% UI)	2021 ASDR, per100,000 people (95% UI)	EAPC (95% UI)
Global	246873.18 (201290.63, 296020.06)	320607.21 (257687.65, 396103.28)	11.26(9.18,13.51)	10.78(8.66, 13.32)	−0.20(−0.27,−0.13)
Andean Latin America	886.90(686.21, 1147.54)	1201.88(946.00, 1514.90)	5.74(4.44, 7.42)	4.44(3.49, 5.59)	−0.85(−1.08,−0.62)
Australasia	2215.39(1413.98,3192.39)	3177.16(2113.75,4391.56)	27.17 (17.34, 39.15)	30.34 (20.19, 41.94)	1.19(0.57, 1.81)
Caribbean	2075.71(1764.98,2473.21)	2278.80(1750.03,2935.02)	13.96 (11.87, 16.64)	12.52(9.61,16.12)	−0.55(−0.75,−0.36)
Central Asia	3860.21(3249.16,4589.93)	5185.93(4178.60,6393.16)	13.57 (11.42, 16.13)	13.87 (11.18, 17.10)	−0.40(−0.65,−0.15)
Central Europe	7739.29(6077.24,9695.77)	5539.77(4112.56,7312.07)	16.52 (12.97, 20.70)	15.82 (11.74, 20.88)	0.33(0.05, 0.60)
Central Latin America	3549.64(3348.53,3797.56)	6273.95(5690.69,6947.43)	5.20(4.91, 5.56)	6.20(5.63, 6.87)	0.97(0.68, 1.27)
Central Sub-Saharan Africa	1581.91(1057.63,2167.86)	4258.52(2844.25,6011.37)	7.62(5.09, 10.44)	7.87(5.26, 11.11)	0.15(0.03, 0.28)
East Asia	26304.42 (18889.24, 32565.92)	16719.21 (13029.40, 20612.21)	4.65(3.34, 5.76)	3.49(2.72, 4.30)	−0.84(−1.10,−0.58)
Eastern Europe	12287.91 (10458.65, 14861.43)	8299.39 (6944.43, 9763.27)	14.33 (12.19, 17.33)	12.54 (10.49, 14.75)	−1.09(−1.53,−0.66)
Eastern Sub-Saharan Africa	4240.23(2759.86,5361.85)	11645.88 (7770.05, 15611.31)	5.98(3.89, 7.56)	6.65(4.44, 8.91)	0.30(0.25, 0.34)
High-income Asia Pacific	6430.80(5135.63,7860.57)	3481.03(2468.47,4645.43)	9.53(7.61, 11.65)	6.89(4.88, 9.19)	−0.60(−1.01,−0.19)
High-income North America	35805.08 (25318.43, 47879.48)	34843.92 (25650.19, 46774.56)	31.60 (22.34, 42.25)	28.29 (20.82, 37.97)	−0.46 (−0.69, −0.24)
North Africa and Middle East	10776.35 (8004.19, 14453.45)	20632.92 (15656.60, 26672.61)	8.05(5.98, 10.80)	8.11(6.16, 10.49)	0.13(0.08, 0.18)
Oceania	217.82(115.04, 325.83)	316.51(212.26, 473.09)	8.20(4.33, 12.27)	5.62(3.77, 8.40)	−1.75(−1.98,−1.52)
South Asia	50144.70 (36670.55, 68422.64)	78102.21 (59243.69, 102557.55)	11.62(8.50,15.85)	9.87(7.49, 12.97)	−0.61(−0.75,−0.46)
Southeast Asia	8574.37(5754.58,10748.54)	10078.36 (7499.89, 12436.95)	4.35(2.92, 5.46)	3.63(2.70, 4.48)	−0.78(−0.87,−0.68)
Southern Latin America	2083.13(1603.88,2604.33)	2388.62(1738.49,3275.62)	10.92(8.41,13.65)	9.26(6.74, 12.70)	−0.43(−0.55,−0.30)
Southern Sub-Saharan Africa	1624.09(1199.89,2077.04)	2314.51(1856.24,2856.72)	7.51(5.55, 9.61)	6.80(5.45, 8.39)	−0.21(−0.86,0.45)
Tropical Latin America	6768.84(6296.86,7393.10)	10908.01 (9883.10, 12092.95)	10.52(9.79,11.50)	12.35 (11.19, 13.69)	0.56(0.30, 0.82)
Western Europe	41840.46 (30598.71, 54091.52)	36175.67 (25773.77, 49995.83)	29.03 (21.23, 37.53)	27.88 (19.86, 38.53)	−0.22(−0.52,0.07)
Western Sub-Saharan Africa	17865.95 (11240.36, 23136.74)	56784.96 (31637.08, 80527.51)	24.96 (15.70, 32.33)	29.70 (16.55, 42.12)	0.60(0.52, 0.67)
SDI					
High SDI	83708.53 (61911.65, 108608.07)	77349.71 (55331.28, 104936.89)	24.13 (17.84, 31.30)	21.90 (15.66, 29.71)	−0.41(−0.61,−0.21)
High-middle SDI	40953.65 (34067.75, 49400.89)	33878.17 (26391.43, 43174.95)	9.05(7.53, 10.92)	7.69(5.99, 9.81)	−0.59(−0.77,−0.42)
Middle SDI	49290.04 (38805.74, 58252.44)	63824.47 (52078.53, 77903.85)	6.55(5.16, 7.74)	6.88(5.61, 8.40)	0.20(0.13, 0.26)
Low-middle SDI	49133.43 (37197.25, 63016.29)	84889.90 (67373.68, 103585.97)	10.84(8.20,13.90)	10.58(8.40,12.91)	−0.17−0.26,−0.09)
Low SDI	23522.36 (15565.58, 31255.57)	60416.36 (40599.89, 75824.19)	12.76(8.45,16.96)	13.45(9.04,16.89)	0.13(0.06, 0.20)

*DALYs* Disability-Adjusted Life Years, *ASDR*: Age-standardized DALYs rate, *UI *Uncertainty interval*, CI *Confidence interval, *SDI *Socio-Demographic Index*, EAPC *Estimated annual percentage change

The global crude incidence and prevalence of AYA IBD in 2021 were 123,343.16 cases (95% UI: 96,841.00 to 158,444.38) and 899,272.10 cases (95% UI: 723,783.54 to 1,128,106.22), respectively. The age-standardized incidence rate (ASIR) and age-standardized prevalence rate (ASPR) were 4.15 per 100,000 (95% UI: 3.26 to 5.33) and 30.23 per 100,000 (95% UI: 24.33 to 37.92), respectively. From 1990 to 2021, the global ASIR and ASPR of AYA IBD changed by an average of 0.33% (95% CI: 0.19 to 0.48) and − 0.15% (95% CI: −0.33 to 0.04) annually, respectively. [Supplementary Tables 2, 3]

The global crude number of deaths due to AYA IBD increased from 2,234.67 cases (95% UI: 1,771.51 to 2,610.35) in 1990 to 2,948.61 cases (95% UI: 2,271.88 to 3,412.62) in 2021. The age-standardized mortality rate (ASMR) remained relatively stable at 0.10 per 100,000 (95% UI: 0.08 to 0.12) in 1990 and 0.10 per 100,000 (95% UI: 0.08 to 0.11) in 2021. The ASMR of AYA IBD decreased by an average of −0.26% annually (95% CI: −0.33 to −0.19) from 1990 to 2021. (Supplementary Table 1).

**Fig. 1:**
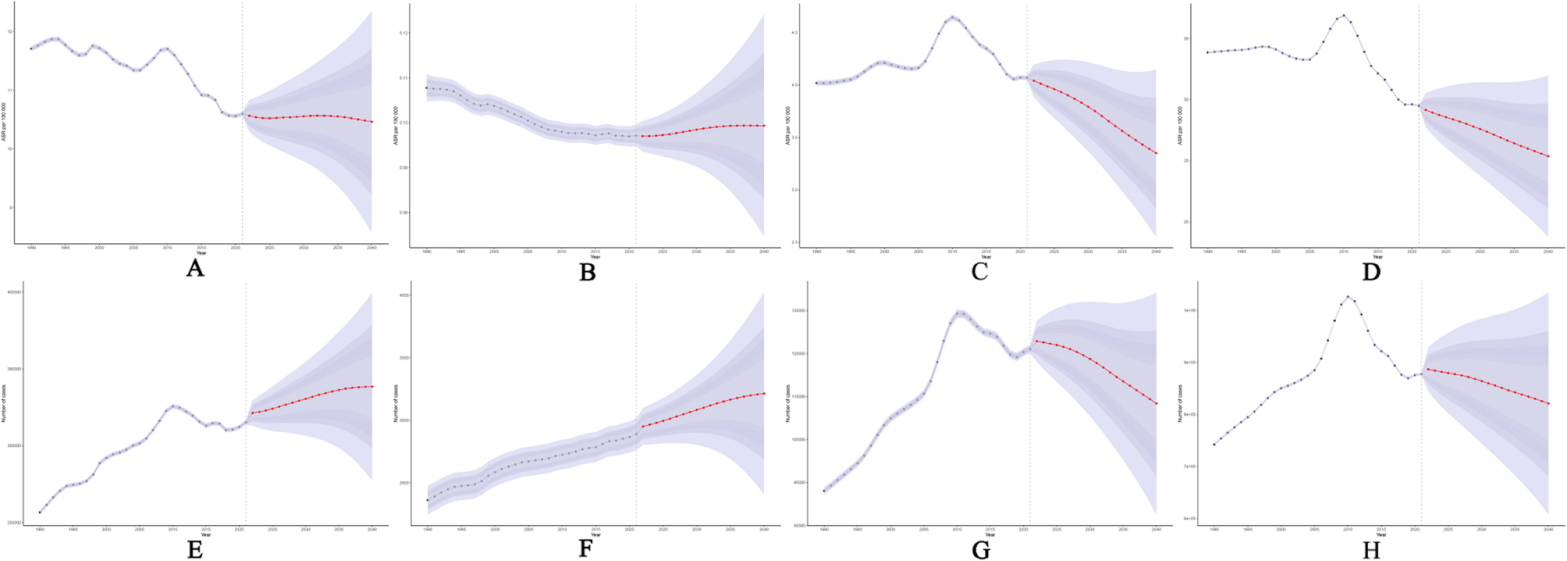
Statistics and predictions of the disease burden of IBD in patients aged 15-39. **A**: ASDR **B**: ASMR **C**: ASIR **D**: ASPR **E**: DALYs **F**: Mortality **G**: Incidence **H**: Prevalence

Overall, ASDR, ASIR, and ASPR showed significant increases around 2005 and substantial declines around 2010. By 2040, the ASDR and ASMR of AYA IBD are projected to remain relatively stable, while ASIR, ASPR, incidence, and prevalence are expected to decline. However, DALYs and the number of deaths are projected to increase. (Fig. 1).

### Regional burden and its trend of AYA IBD from 1990 to 2021

In 2021, among the 21 GBD regions, the highest ASDR of AYA IBD was in Australasia, at 30.34 DALYs per 100,000 (95% UI: 20.19 to 41.94), followed by Western Sub-Saharan Africa, at 29.70 DALYs per 100,000 (95% UI: 16.55 to 42.12).In 2021, while the lowest was in East Asia, at 3.49 DALYs per 100,000 (95% UI: 2.72 to 4.30). During 1990–2021, Australasia experienced the largest increase in ASDR, with an average annual increase of 1.19% (95% CI: 0.57 to 1.81), while Oceania had the largest decrease, with an average annual decline of −1.75% (95% CI: −1.98 to −1.52). (Table 1).

In 2021, the highest ASIR was in Australasia, at 22.96 per 100,000 (95% UI: 18.26 to 28.85), while the lowest was in Central Latin America, at 0.55 per 100,000 (95% UI: 0.42 to 0.73). During 1990–2021, ASIR increased in 20 of the 21 GBD regions, with the largest increase in East Asia, at an average annual rate of 3.28% (95% CI: 2.58 to 3.99). The only region with a decline was High-income North America, with an average annual decrease of −0.14% (95% CI: −0.34 to 0.05), ranking second highest in ASIR among all regions. [Supplementary Table 2]

In 2021, three regions had an ASPR greater than 100 per 100,000: Australasia, Western Europe, and High-income North America. Australasia had the highest ASPR, at 178.16 per 100,000 (95% UI: 142.23 to 226.13), while Central Latin America had the lowest, at 4.56 per 100,000 (95% UI: 3.47 to 5.93). During 1990–2021, East Asia had the largest increase in ASPR, at 2.60% (95%CI: 1.81 to 3.39) per year. In contrast, the ASPR decreased in four regions, among which the largest decreases were observed in High-income North America and Western Europe, at −0.95% (95% CI: −1.24 to −0.66) and − 0.25% (95% CI: −0.56 to 0.06) per year, respectively. [Supplementary Table 3]

In 2021, the highest ASMR was in Western Sub-Saharan Africa, at 0.46 per 100,000 (95% UI: 0.24 to 0.67), while the lowest was in High-income Asia Pacific, at 0.03 per 100,000 (95% UI: 0.03 to 0.04). During 1990–2021, Australasia had the largest increase in ASMR, at 2.33% (95% CI: 1.40 to 3.26) per year, High-income Asia Pacific had the largest decrease in ASMR, at −3.69% (95% CI: −3.96 to −3.42), while Western Europe remained almost unchanged, with a rate of 0.00% (95% CI: −0.33 to 0.34) per year. [Supplementary Table 1]

### Burden and its trend of AYA IBD from 1990 to 2021 by nation and SDI

**Fig. 2:**
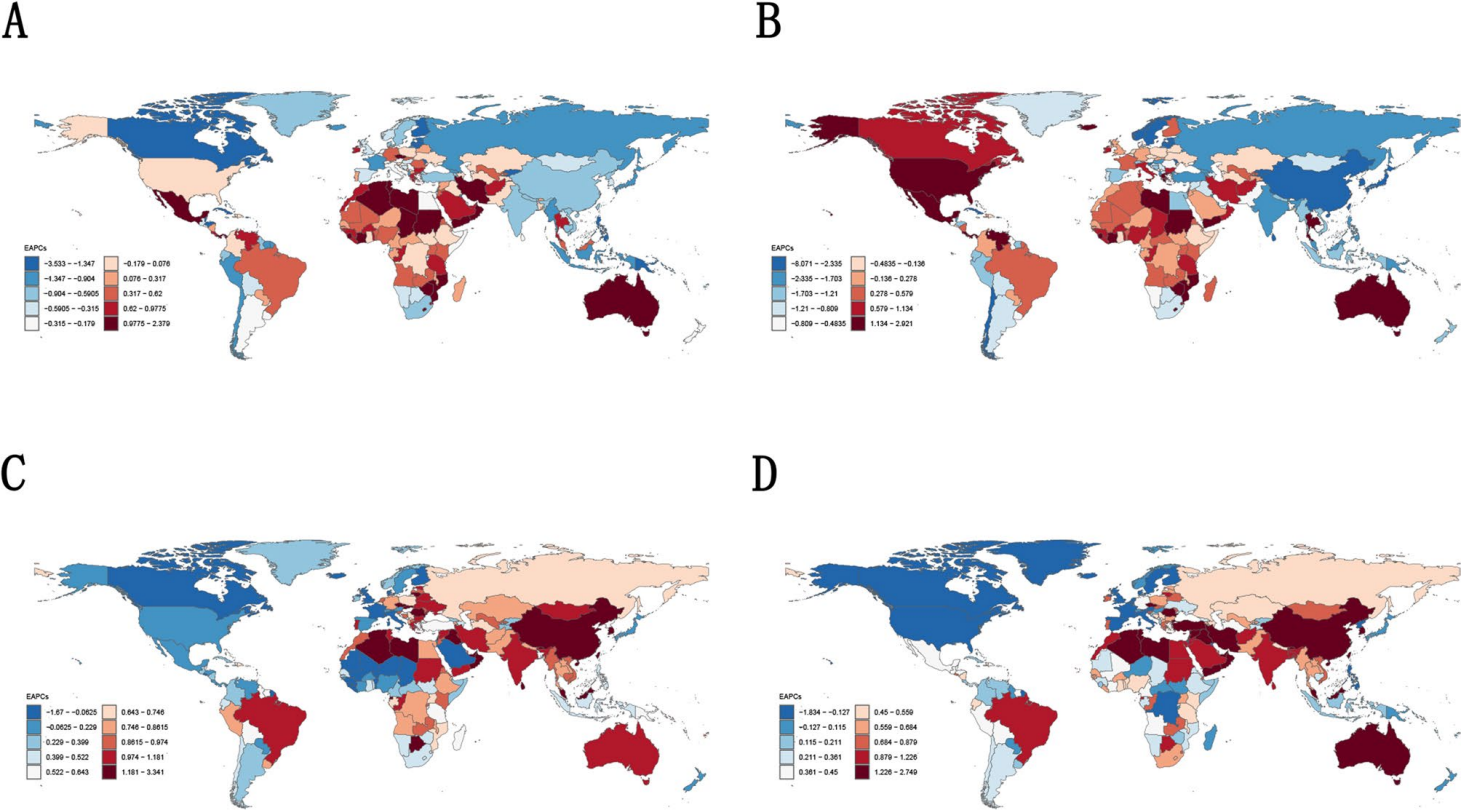
EAPC analysis of age-standardized incidence, prevalence, DALYs and mortality rates of IBD in patients aged 15–39 from 1990–2021 **A**: ASDR **B**: ASMR **C**: ASIR **D**: ASPR

During 1990–2021, Libya had the largest average annual increase in ASDR, at 2.38% (95% CI: 2.12 to 2.64), while American Samoa had the largest decrease, at −4.96% (95% CI: −5.67 to −4.25) per year. (Fig. 2).

During 1990–2021, China, Libya, Taiwan (Province of China), and the Republic of Korea had the largest increases in ASIR, while Libya, China, the Republic of Korea, and Taiwan (Province of China) had the largest increases in ASPR. In contrast, only 21 and 38 countries or territories experienced a decline in ASIR and ASPR respectively. Finland, Denmark, Iceland, and Canada had the largest decreases in ASIR, while Canada, Finland, France, and Iceland had the largest decreases in ASPR. (Fig. 2).

Compared to 1990, Australia had the largest increase in ASMR, at 2.92 per 100,000 (95% UI: 1.92 to 3.94), while Estonia had the largest decrease, at −8.07 per 100,000 (95% UI: −9.85 to −6.26). (Fig. 2).

High SDI countries had the highest ASDR, ASIR, and ASPR in both 1990 and 2021. Furthermore, in 2021, the ASDR, ASIR, and ASPR of High SDI countries all showed substantial gaps compared with those of other countries. The highest ASMR in 1990 and 2021 was in Low SDI countries. During 1990–2021, the largest increase in ASMR was also in Low SDI countries, while the largest increases in ASDR, ASIR, and ASPR were in Middle SDI countries. The largest decreases in ASDR and ASMR were in High-middle SDI countries, while the smallest increases in ASIR and the largest decreases in ASPR were observed in high SDI countries. (Table 1, Supplementary Tables 1, 2, 3).

### Correlations between EAPC and ASR as well as SDI

**Fig. 3:**
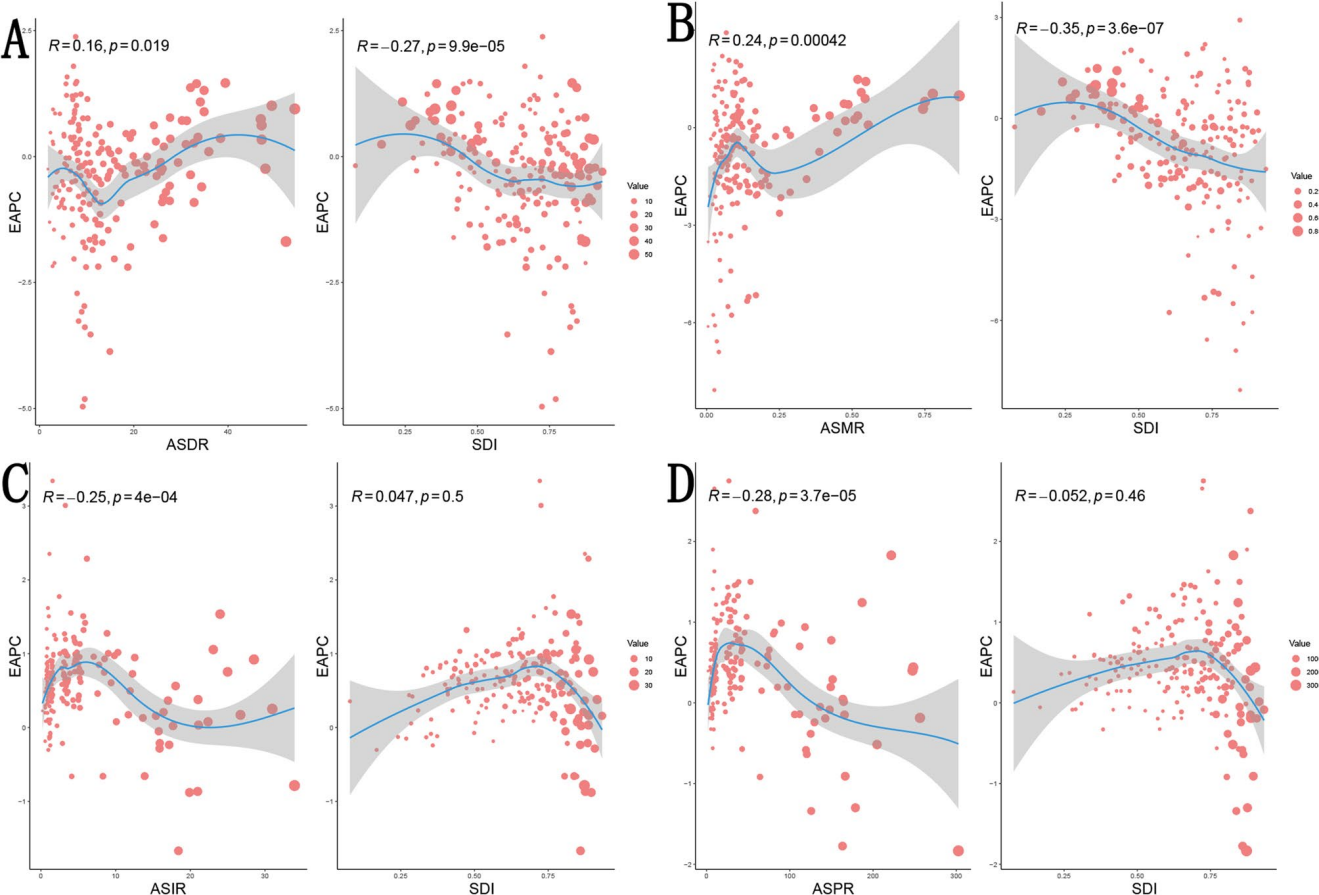
The correlations between EAPC and ASR as well as SDI in patients aged 15-39. **A**: ASDR **B**: ASMR **C**: ASIR **D**: ASPR

We conducted correlation analysis to determine potential correlations between EAPC and ASR as well as SDI. The EAPC of ASDR was positively correlated with ASDR (*R* = 0.16, *p* = 0.019) and negatively correlated with SDI (*R* = −0.27, *p* = 9.9e-05). The EAPC of ASMR was negatively correlated with ASMR (*R* = −0.24, *p* = 0.00042) and SDI (*R* = −0.35, *p* = 3.6e-07). Meanwhile, ASIR and ASPR were negatively correlated with their respective EAPCs (*R* = −0.25, *p* = 4e-04) and (*R* = −0.28, *p* = 3.7e-05), respectively. However, neither ASIR nor ASPR EAPCs showed significant correlations with SDI (*R* = 0.047, *p* = 0.5) and (*R* = −0.052, *p* = 0.46), respectively. (Fig. 3).

### Global burden of AYA IBD by sex and 5-year age group

**Fig. 4:**
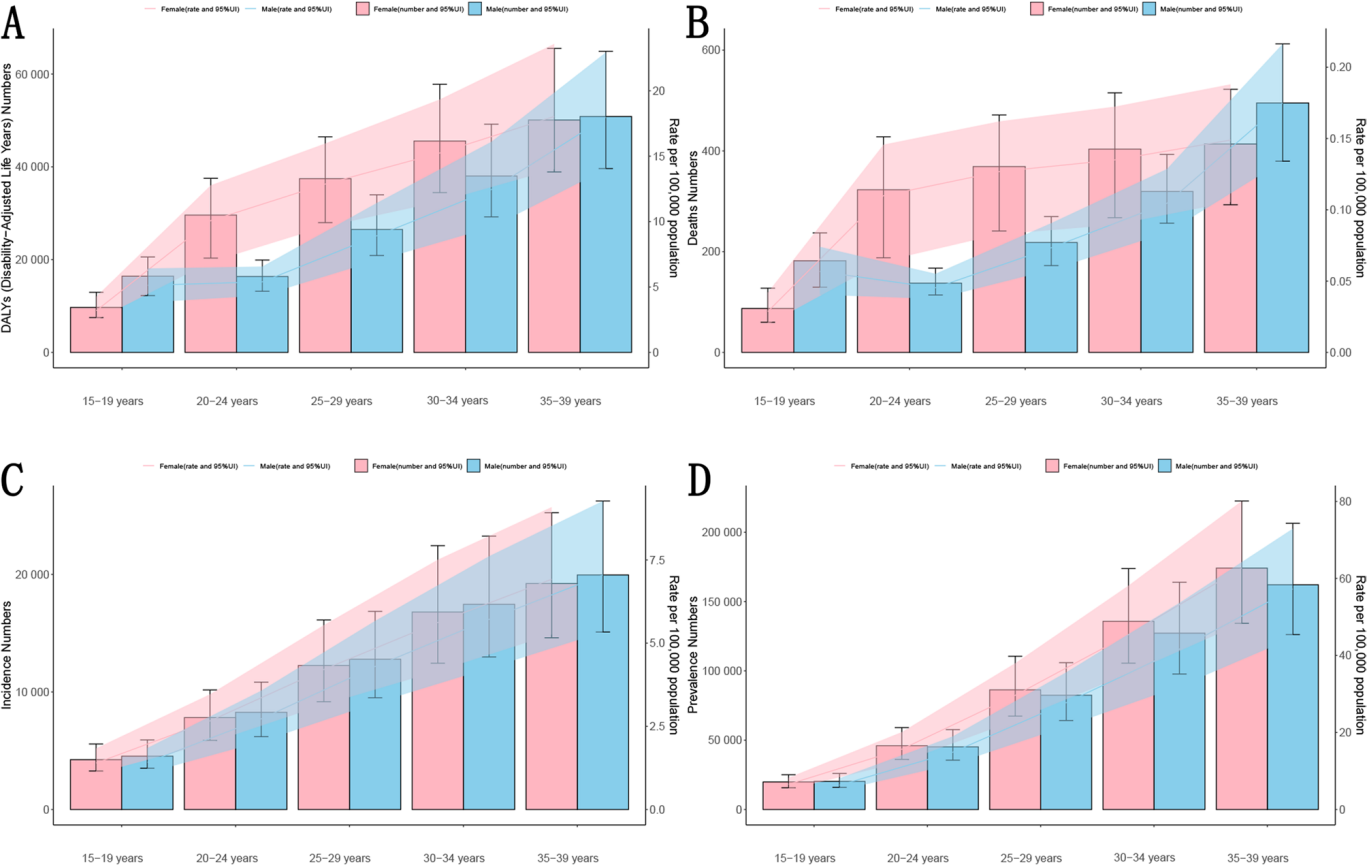
Age and sex distribution of age-standardized incidence, prevalence, DALYs and mortality rates of IBD in patients aged 15-39 in 2021 globally. **A**: ASDR **B**: ASMR **C**: ASIR **D**: ASPR

In 2021, female patients with AYA IBD exhibited higher DALYs, number of deaths, number of prevalence cases, and their respective ASR compared to males. However, number of incidence cases and ASIR were higher in males. In further age stratification, except for males aged 20–24 years, who had lower DALYs, number of deaths, and ASMR than those aged 15–19 years, the disease burden increased with age, with the highest burden in the 35–39 age group. (Fig. 4).

Females had higher DALYs, number of prevalence cases, number of deaths, and corresponding ASR in all age groups except 15–19 years and 35–39 years. However, number of incidence cases and ASIR were lower in females across all age groups. (Fig. 4).

## Discussion

Compared to GBD 2019, the updated GBD 2021 shows differences in data; therefore, it is necessary to use the latest data for investigation. In this study, we comprehensively investigated the burden of AYA IBD from 1990 to 2021 based on the latest GBD database, from global, regional, national, SDI, gender, and age perspectives [13, 15]. The results indicate that during 1990–2021, the DALYs, number of deaths, number of incidence cases, number of prevalence cases, and ASIR of global AYA IBD showed an increasing trend. Moreover, DALYs and number of deaths are projected to continue rising steadily over the next 20 years, highlighting the significant disease burden of AYA IBD poses to society and healthcare systems. Further analysis revealed significant differences in the disease burden of AYA IBD across different countries.

Traditionally, IBD has been considered as a common disease in Western developed countries. We found that in 2021, the ASDR, ASIR, and ASPR of AYA IBD in Australasia, Western Europe, and High-income North America were significantly higher than in most other regions, consistent with previous reports [9, 16]. It is encouraging that the ASDR in High SDI and High-middle SDI countries showed a downward trend, likely due to the advent of new drugs such as biologics, improvements in surgical techniques, the improvement of nursing standards and other factors that have improved the quality of life for AYA IBD patients [17–20]. However, in some developed countries such as Australia, Slovakia, and Germany, the ASDR of AYA IBD in 2021 was higher than in 1990, possibly due to poor familiarity with and adherence to guidelines, as well as insufficient mental health support for patients [21].

Meanwhile, the ASIR and ASPR of AYA IBD in Middle SDI countries are increasing. This may be due to the rapid economic development and significant improvement in living standards, which have also led to changes in diet habits such as high-fat, high-sugar, and ultra-processed Western dietary patterns [22, 23]. Moreover - with the acceleration of urbanization - lack of exercise, and disrupted sleep schedule have become more common in developing countries. The increase in the number of individuals with genetic susceptibility during globalization is also an important factor [23–25]. Additionally, with economic development, the diagnostic capabilities for IBD have significantly improved, including deeper disease understanding, advancements in endoscopic techniques, and the widespread adoption of calprotectin testing. These factors have collectively enabled the identification of previously undiagnosed cases of AYA IBD [18, 26].

In light of regional variations in disease burden, the following recommendations should be considered for implementation. In regions and countries with high SDI and better medical conditions for AYA IBD, it is essential to develop more rational prevention and management policies and seek better treatment options, such as Artificial intelligence and next-generation medicine [27, 28]. After all, current treatments, represented by biologics, still have therapeutic limits and may increase the risk of certain malignancies [19, 29, 30]. In regions with scarce medical resources and rapidly increasing prevalence, especially in middle SDI countries, it is imperative to train more IBD specialists, upgrade and expand the coverage of medical infrastructure, strengthen public-oriented publicity and education (e.g., public science programs focused on early symptoms), and develop comprehensive health insurance systems to improve the affordability of AYA IBD treatment. Moreover, policymakers worldwide should actively engage in international cooperation, with developed countries obtaining more research data and developing countries learning advanced disease management and prevention methods from developed countries.

As is well known, age is an important factor affecting the burden of IBD [2]. In our age-stratified analysis, except for AYA IBD patients aged 20–24 years in males, who had lower DALYs, number of deaths, and ASMR than those aged 15–19 years, the disease burden of AYA IBD increased by age, with the highest burden in the 35–39 age group. This may be due to increasing social responsibilities and pressures, more incidence of various related complications, longer course of disease, and higher difficulty of treatment [31–33]. Therefore, with the advent of an aging society, policymakers may need to allocate additional healthcare resources to meet the needs of AYA IBD patients.

Although previous studies have reported no significant differences in global IBD between males and females [9]. Our analysis of AYA IBD in different age groups shows more specific gender differences in 2021. ASDR and ASPR were significantly higher in females than in males, possibly because females aged 15–39 years are in the peak secretion period of female sex hormones, including 17β-estradiol, progesterone, and luteinizing hormone, as well as in the peak childbearing period, both of which pose greater challenges for disease monitoring and management [4, 34, 35]. It is worth noting that in China, the ASPR of male AYA IBD is lower than that of females, while the ASDR remains significantly higher in males. This phenomenon may be attributable to the more severe IBD symptoms experienced by males due to smoking, such as premature mortality, which could be one of the contributing factors to the higher ASDR observed in Chinese males [19, 36, 37]. Therefore, regulating sex hormones in female patients might provide new ideas for treatment while decreasing smoking prevalence should be prioritized by policymakers. Additionally, some studies suggest that hormones may affect the brain-gut-microbiota axis, and certain genetic loci associated with IBD are located on the X chromosome, which are also reasons for the different burdens of AYA IBD between males and females. However, the underlying mechanisms remain unclear and require further basic research [1, 38, 39].

This study also has some limitations. First, the underdeveloped healthcare systems in Low and Low-middle SDI countries, marked by inadequate hospital facilities, a paucity of specialist healthcare workers, limited medical financial resources, and fragmented healthcare security frameworks, can result in potential misdiagnosis and missed diagnosis of AYA IBD, thereby leading to an underestimation of case numbers. Second, the data collection process involves IBD departments in different countries and regions, which may lead to differences in diagnostic criteria and inherent biases in our study data. Third, the inability to distinguish between CD and UC limits our ability to capture epidemiological differences between these two forms of IBD. This limitation is particularly critical for research focusing on adolescent and young adult populations. Given that CD has a significantly higher incidence than UC among adolescents and young adults, aggregate disease burden estimates based on undifferentiated data may substantially underestimate the true disease burden of CD within this demographic and impede the formulation of optimal prevention and treatment recommendation. Finally, with the emergence of new therapies, data on their safety for specific populations, such as children or women of childbearing age, are still lacking.

## Conclusion

The results of this study show that AYA IBD poses a significant global disease burden, and DALYs are projected to continue rising through 2040. In 2021, high SDI countries and regions still bear a heavy burden of AYA IBD. In Middle SDI countries, the incidence and prevalence of AYA IBD are increasing rapidly. Moreover, there are differences in the disease burden across different age groups and genders. Therefore, it is urgent for policymakers to prioritize understanding the prevention, management, and treatment of AYA IBD, focusing on closely related factors to develop effective measures and allocate resources more reasonably.

## Supplementary Information

Supplementary Material 1.

Supplementary Material 2.

Supplementary Material 3.

## Data Availability

The data used in this study are available from the GBD 2021 (http://ghdx.healthdata.org/gbd-results-tool).

## Abbreviations

IBD: Inflammatory bowel disease
AYA: Adolescents and young adults
UI: Uncertainty interval
CI: Confidence interval
CD: Crohn’s disease
UC: Ulcerative colitis
DALYs: Disability-adjusted life years
GBD: Global burden of disease
SDI: Socio-demographic index
GHDx: Global health data exchange
BAPC: Bayesian age-period-cohort
INLA: Integrated nested laplace approximation
ASR: Age-standardized rate
ASPR: Age-standardized prevalence rate
ASDR: Age-standardized DALYs rate
ASIR: Age-standardized incidence rate
ASMR: Age-standardized mortality rate
EAPC: Estimated annual percentage change
