# Supplementary material for: Analysis of global burden of inflammatory bowel disease among adolescents and young adults from 1990 to 2021 and projections to 2040
Source: BMC Public Health. 2025 Sep 24;25:3087. doi: 10.1186/s12889-025-24105-0 (PMC12462196; doi:10.1186/s12889-025-24105-0)
Supplement: Supplementary file 1 — Supplementary Material 1. [file 12889_2025_24105_MOESM1_ESM.docx]

**Supplementary Table 1. Summary of IBD in patients aged 15-39 mortality and age-standardized mortality rates in 1990 and 2021.**

|  | 1990 cases  (95% UI) | 2021 cases  (95% UI) | 1990 ASMR,  per 100,000 people  (95% UI) | 2021 ASMR,  per 100,000 people  (95% UI) | EAPC  (95% UI) |
| --- | --- | --- | --- | --- | --- |
| Global | 2234.67(1771.51,2610.35) | 2948.61(2271.88,3412.62) | 0.10(0.08,  0.12) | 0.10(0.08,  0.11) | -0.26(-0.33,-0.19) |
| Andean Latin America | 10.09(7.42,  13.75) | 11.52(8.61,  15.56) | 0.07(0.05,  0.09) | 0.04(0.03,  0.06) | -1.37(-1.65,-1.09) |
| Australasia | 2.91(2.68,3.17) | 6.35(5.62,7.16) | 0.04(0.03,  0.04) | 0.06(0.05,  0.07) | 2.33(1.40,  3.26) |
| Caribbean | 25.34(21.08,  30.90) | 25.86(17.87,  36.16) | 0.17(0.14,  0.21) | 0.14(0.10,  0.20) | -0.81(-1.08,-0.55) |
| Central Asia | 39.14(34.67,  43.85) | 48.96(40.55,  58.81) | 0.14(0.12,  0.15) | 0.13(0.11,  0.16) | -0.87(-1.21,-0.53) |
| Central Europe | 53.64(50.21,  60.01) | 28.83(26.26,  31.68) | 0.11(0.11,  0.13) | 0.08(0.07,  0.09) | -0.65(-0.88,-0.42) |
| Central Latin America | 50.81(49.18,  52.75) | 92.95(84.09,  102.87) | 0.07(0.07,  0.08) | 0.09(0.08,  0.10) | 1.13(0.79,  1.48) |
| Central Sub-Saharan Africa | 20.49(12.11,  29.84) | 55.84(32.31,  85.39) | 0.10(0.06,  0.14) | 0.10(0.06,  0.16) | 0.24(0.11,  0.38) |
| East Asia | 349.33(231.52,  453.45) | 162.98(129.27,  213.12) | 0.06(0.04,  0.08) | 0.03(0.03,  0.04) | -2.48(-2.76,-2.20) |
| Eastern Europe | 149.80(135.21,  185.86) | 91.00(81.47,  99.61) | 0.17(0.16,  0.22) | 0.14(0.12,  0.15) | -1.70(-2.24,-1.16) |
| Eastern Sub-Saharan Africa | 55.17(31.85,  71.97) | 151.29(90.19,  211.64) | 0.08(0.04,  0.10) | 0.09(0.05,  0.12) | 0.31(0.25,  0.37) |
| High-income Asia Pacific | 58.97(44.39,  70.51) | 14.93(12.67,  19.87) | 0.09(0.07,  0.10) | 0.03(0.03,  0.04) | -3.69(-3.96,-3.42) |
| High-income North America | 102.64(100.04,  105.80) | 156.42(149.61,  162.54) | 0.09(0.09,  0.09) | 0.13(0.12,  0.13) | 1.45(1.28,  1.62) |
| North Africa and Middle East | 89.92(61.33,  143.68) | 130.13(101.71,  183.18) | 0.07(0.05,  0.11) | 0.05(0.04,  0.07) | -0.99(-1.14,-0.84) |
| Oceania | 3.34(1.62,5.16) | 4.60(2.84,7.23) | 0.13(0.06,  0.19) | 0.08(0.05,  0.13) | -1.98(-2.23,-1.72) |
| South Asia | 545.54(351.44,  799.57) | 656.64(482.38,  925.14) | 0.13(0.08,  0.19) | 0.08(0.06,  0.12) | -1.66(-1.85,-1.46) |
| Southeast Asia | 110.51(67.02,  141.34) | 121.74(83.01,  157.03) | 0.06(0.03,  0.07) | 0.04(0.03,  0.06) | -1.09(-1.22,-0.96) |
| Southern Latin America | 15.71(14.53,  16.88) | 11.58(10.80,  12.45) | 0.08(0.08,  0.09) | 0.04(0.04,  0.05) | -1.60(-1.90,-1.31) |
| Southern Sub-Saharan Africa | 21.52(14.85,  27.56) | 28.28(21.78,  35.77) | 0.10(0.07,  0.13) | 0.08(0.06,  0.11) | -0.37(-1.21,0.48) |
| Tropical Latin America | 93.47(89.76,  98.02) | 144.14(136.98,  150.59) | 0.15(0.14,  0.15) | 0.16(0.16,  0.17) | 0.48(0.13,  0.83) |
| Western Europe | 160.37(153.91,  167.41) | 128.91(123.00,  135.04) | 0.11(0.11,  0.12) | 0.10(0.09,  0.10) | 0.00(-0.33,0.34) |
| Western Sub-Saharan Africa | 275.97(166.04,  363.18) | 875.66(460.65,  1274.08) | 0.39(0.23,  0.51) | 0.46(0.24,  0.67) | 0.60(0.52,  0.68) |
| SDI |  |  |  |  |  |
| High SDI | 347.11(325.45,  361.12) | 316.43(307.73,  326.18) | 0.10(0.09,  0.10) | 0.09(0.09,  0.09) | -0.14(-0.26,-0.03) |
| High-middle SDI | 385.10(350.80,  437.99) | 251.78(231.75,  289.67) | 0.09(0.08,  0.10) | 0.06(0.05,  0.07) | -1.75(-1.97,-1.53) |
| Middle SDI | 600.86(457.60,  707.74) | 652.86(522.81,  778.07) | 0.08(0.06,  0.09) | 0.07(0.06,  0.08) | -0.55(-0.67,-0.44) |
| Low-middle SDI | 579.20(392.40,  775.90) | 909.58(654.91,  1098.44) | 0.13(0.09,  0.17) | 0.11(0.08,  0.14) | -0.61(-0.72,-0.50) |
| Low SDI | 320.07(192.29,  438.47) | 815.91(505.38,  1079.00) | 0.17(0.10,  0.24) | 0.18(0.11,  0.24) | 0.07(-0.00,0.15) |

**ASMR: Age-standardized mortality rate，UI: Uncertainty interval，CI: Confidence interval，SDI: Socio-Demographic Index，EAPC: Estimated annual percentage change**
