# Supplementary material for: Analysis of global burden of inflammatory bowel disease among adolescents and young adults from 1990 to 2021 and projections to 2040
Source: BMC Public Health. 2025 Sep 24;25:3087. doi: 10.1186/s12889-025-24105-0 (PMC12462196; doi:10.1186/s12889-025-24105-0)
Supplement: Supplementary file 2 — Supplementary Material 2. [file 12889_2025_24105_MOESM2_ESM.docx]

**Supplementary Table 2. Summary of IBD in patients aged 15-39 incidence and age-standardized incidence rates in 1990 and 2021.**

|  | 1990 cases  (95% UI) | 2021 cases  (95% UI) | 1990 ASIR,  per 100,000 people  (95% UI) | 2021 ASIR,  per 100,000 people  (95% UI) | EAPC  (95% UI) |
| --- | --- | --- | --- | --- | --- |
| Global | 84960.65  (68688.97,  104858.22) | 123343.16  (96841.00,  158444.38) | 3.88(3.13,  4.78) | 4.15(3.26,  5.33) | 0.33(0.19,  0.48) |
| Andean Latin America | 186.23(143.57,  243.34) | 396.32(298.88,  530.02) | 1.20(0.93,  1.57) | 1.46(1.10,  1.96) | 0.67(0.61,  0.74) |
| Australasia | 1670.22(1322.97,2155.72) | 2403.68(1911.80,3021.21) | 20.48  (16.23,  26.44) | 22.96  (18.26,  28.85) | 0.90(0.48,  1.32) |
| Caribbean | 370.66(291.28,  476.06) | 544.57(421.35,  712.02) | 2.49(1.96,  3.20) | 2.99(2.31,  3.91) | 0.47(0.37,  0.56) |
| Central Asia | 1128.28(886.77,  1450.33) | 1889.36(1469.30,2466.25) | 3.97(3.12,  5.10) | 5.05(3.93,  6.60) | 0.68(0.56,  0.80) |
| Central Europe | 2968.72(2407.32,3701.03) | 2748.18(2158.43,3505.38) | 6.34(5.14,  7.90) | 7.85(6.16,  10.01) | 1.06(0.78,  1.35) |
| Central Latin America | 366.30(276.75,  480.92) | 553.37(422.02,  737.43) | 0.54(0.41,  0.70) | 0.55(0.42,  0.73) | 0.24(0.08,  0.40) |
| Central Sub-Saharan Africa | 249.87(194.70,  324.06) | 821.08(636.67,  1074.70) | 1.20(0.94,  1.56) | 1.52(1.18,  1.99) | 0.79(0.76,  0.82) |
| East Asia | 4159.89(3199.78,5526.66) | 7401.03(5673.48,9990.82) | 0.74(0.57,  0.98) | 1.54(1.18,  2.09) | 3.28(2.58,  3.99) |
| Eastern Europe | 2731.77(2114.30,3597.08) | 2707.33(2068.54,3633.30) | 3.19(2.47,  4.19) | 4.09(3.13,  5.49) | 0.85(0.68,  1.03) |
| Eastern Sub-Saharan Africa | 786.69(612.16,  1023.17) | 2336.07(1818.61,3064.91) | 1.11(0.86,  1.44) | 1.33(1.04,  1.75) | 0.72(0.65,  0.79) |
| High-income Asia Pacific | 1738.01(1384.17,2209.46) | 1702.41(1325.56,2179.80) | 2.58(2.05,  3.27) | 3.37(2.62,  4.31) | 1.31(0.60,  2.03) |
| High-income North America | 22174.58  (18313.64,  26883.07) | 23715.32  (18906.78,  29303.57) | 19.57  (16.16,  23.72) | 19.25  (15.35,  23.79) | -0.14(-0.34,0.05) |
| North Africa and Middle East | 3860.84(3048.66,4916.56) | 9149.32(7093.45,11884.38) | 2.88(2.28,  3.67) | 3.60(2.79,  4.67) | 0.87(0.81,  0.93) |
| Oceania | 16.93(12.97,  22.41) | 42.64(32.66,  57.83) | 0.64(0.49,  0.84) | 0.76(0.58,  1.03) | 0.48(0.45,  0.52) |
| South Asia | 15946.09  (12325.70,  20777.30) | 37046.86  (28602.29,  48809.15) | 3.69(2.86,  4.81) | 4.68(3.62,  6.17) | 0.92(0.75,  1.10) |
| Southeast Asia | 1194.85(923.02,  1570.44) | 2088.41(1605.15,2777.66) | 0.61(0.47,  0.80) | 0.75(0.58,  1.00) | 0.69(0.66,  0.73) |
| Southern Latin America | 882.67(683.98,  1162.75) | 1336.27(1035.74,1769.57) | 4.63(3.58,  6.09) | 5.18(4.02,  6.86) | 0.35(0.29,  0.40) |
| Southern Sub-Saharan Africa | 286.28(222.68,  374.17) | 517.29(399.77,  678.16) | 1.32(1.03,  1.73) | 1.52(1.17,  1.99) | 0.49(0.37,  0.62) |
| Tropical Latin America | 1053.56(819.53,  1384.24) | 2210.86(1702.07,2979.02) | 1.64(1.27,  2.15) | 2.50(1.93,  3.37) | 0.97(0.34,  1.61) |
| Western Europe | 22388.06  (18835.24,  26357.98) | 21445.74  (17090.41,  26749.36) | 15.53  (13.07,  18.29) | 16.53  (13.17,  20.61) | 0.15(-0.08,0.37) |
| Western Sub-Saharan Africa | 800.15(618.21,  1040.18) | 2287.05(1776.35,2963.98) | 1.12(0.86,  1.45) | 1.20(0.93,  1.55) | 0.11(0.04,  0.19) |
| SDI |  |  |  |  |  |
| High SDI | 45103.32  (37452.76,  54473.98) | 48202.88  (38440.86,  59637.79) | 13.00  (10.79,  15.70) | 13.65  (10.88,  16.88) | 0.08(-0.09,0.25) |
| High-middle SDI | 13089.70  (10479.95,  16509.02) | 15596.09  (12202.37,  20398.40) | 2.89(2.32,  3.65) | 3.54(2.77,  4.63) | 0.96(0.67,  1.25) |
| Middle SDI | 10425.47  (8085.29,  13728.08) | 21597.63  (16636.74,  28354.40) | 1.39(1.07,  1.82) | 2.33(1.79,  3.06) | 1.97(1.71,  2.23) |
| Low-middle SDI | 12711.77  (9846.25,  16524.03) | 28051.22  (21730.35,  36696.64) | 2.80(2.17,  3.64) | 3.50(2.71,  4.57) | 0.82(0.69,  0.94) |
| Low SDI | 3545.91(2717.68,4605.26) | 9802.16(7598.99,12803.60) | 1.92(1.47,  2.50) | 2.18(1.69,  2.85) | 0.55(0.46,  0.64) |

**ASIR: Age-standardized incidence rate，UI: Uncertainty interval，CI: Confidence interval，SDI: Socio-Demographic Index，EAPC: Estimated annual percentage change**
