# Supplementary material for: Analysis of global burden of inflammatory bowel disease among adolescents and young adults from 1990 to 2021 and projections to 2040
Source: BMC Public Health. 2025 Sep 24;25:3087. doi: 10.1186/s12889-025-24105-0 (PMC12462196; doi:10.1186/s12889-025-24105-0)
Supplement: Supplementary file 3 — Supplementary Material 3. [file 12889_2025_24105_MOESM3_ESM.docx]

**Supplementary Table 3. Summary of IBD in patients aged 15-39 prevalence and age-standardized prevalence rates in 1990 and 2021.**

|  | 1990 cases  (95% UI) | 2021 cases  (95% UI) | 1990 ASPR,  per 100,000 people  (95% UI) | 2021 ASPR,  per 100,000 people  (95% UI) | EAPC  (95% UI) |
| --- | --- | --- | --- | --- | --- |
| Global | 705148.95  (586377.42,  859667.32) | 899272.10  (723783.54,  1128106.22) | 32.17  (26.75,  39.22) | 30.23  (24.33,  37.92) | -0.15(-0.33,0.04) |
| Andean Latin America | 1518.09(1193.38,1936.97) | 3043.96(2359.41,3943.13) | 9.82(7.72,  12.53) | 11.24(8.71,14.56) | 0.40(0.28,  0.52) |
| Australasia | 13459.29  (10866.07,  17189.60) | 18655.40  (14892.92,  23677.73) | 165.07  (133.26,  210.82) | 178.16  (142.23,  226.13) | 1.09(0.44,  1.75) |
| Caribbean | 3295.64(2645.77,4117.62) | 4440.99(3558.71,5541.79) | 22.17  (17.80,  27.70) | 24.40  (19.55,  30.44) | 0.13(0.04,  0.22) |
| Central Asia | 8944.06(7104.33,11375.89) | 13900.38  (11117.39,  17782.63) | 31.43  (24.97,  39.98) | 37.18  (29.74,  47.56) | 0.41(0.24,  0.57) |
| Central Europe | 28801.39  (23706.26,  35556.98) | 24528.18  (19659.28,  30649.83) | 61.48  (50.60,  75.90) | 70.04  (56.14,  87.52) | 0.88(0.53,  1.24) |
| Central Latin America | 2932.69(2246.56,3779.55) | 4608.48(3508.45,5998.36) | 4.30(3.29,  5.54) | 4.56(3.47,  5.93) | 0.24(0.11,  0.37) |
| Central Sub-Saharan Africa | 1834.36(1462.41,2304.84) | 4909.68(3898.55,6316.20) | 8.84(7.04,  11.10) | 9.08(7.21,  11.68) | -0.07(-0.20,0.06) |
| East Asia | 31185.65  (24553.58,  39690.17) | 44405.86  (34806.04,  57262.87) | 5.51(4.34,  7.02) | 9.27(7.27,  11.95) | 2.60(1.81,  3.39) |
| Eastern Europe | 22294.40  (17718.25,  28288.11) | 19750.82  (15389.92,  25649.82) | 25.99  (20.66,  32.98) | 29.85  (23.26,  38.76) | 0.49(0.23,  0.76) |
| Eastern Sub-Saharan Africa | 5224.06(4162.85,6633.37) | 14733.10  (11764.15,  18628.03) | 7.37(5.87,  9.36) | 8.41(6.72,  10.63) | 0.37(0.33,  0.42) |
| High-income Asia Pacific | 17548.47  (14214.93,  21869.37) | 16589.65  (13196.21,  20703.86) | 26.00  (21.06,  32.40) | 32.83  (26.11,  40.97) | 1.34(0.56,  2.13) |
| High-income North America | 194089.27  (164868.70,  234580.80) | 170008.61  (136993.09,  212977.70) | 171.28  (145.50,  207.02) | 138.01  (111.21,  172.89) | -0.95(-1.24,-0.66) |
| North Africa and Middle East | 31253.94  (25393.29,  39210.77) | 78194.72  (62130.38,  99424.46) | 23.35  (18.97,  29.30) | 30.75  (24.44,  39.10) | 1.20(1.08,  1.31) |
| Oceania | 138.29(107.26,  178.23) | 291.47(224.83,  376.41) | 5.21(4.04,  6.71) | 5.17(3.99,  6.68) | -0.14(-0.20,-0.08) |
| South Asia | 100341.33  (79713.78,  128940.02) | 230730.79  (182319.28,  298587.59) | 23.25  (18.47,  29.87) | 29.17  (23.05,  37.75) | 0.97(0.78,  1.16) |
| Southeast Asia | 10289.85  (8138.46,  13027.26) | 15877.59  (12327.32,  20329.08) | 5.22(4.13,  6.61) | 5.73(4.45,  7.33) | 0.43(0.37,  0.49) |
| Southern Latin America | 7217.54(5666.58,9188.60) | 10843.96  (8417.16,  14048.26) | 37.83  (29.70,  48.16) | 42.04  (32.63,  54.46) | 0.29(0.25,  0.32) |
| Southern Sub-Saharan Africa | 1906.53(1521.38,2403.92) | 3764.84(2966.30,4735.03) | 8.82(7.04,  11.12) | 11.06(8.72,13.91) | 0.58(0.49,  0.68) |
| Tropical Latin America | 7044.69(5547.97,8853.87) | 14538.00  (11353.08,  18942.60) | 10.95(8.63,13.77) | 16.46  (12.86,  21.45) | 0.99(0.43,  1.56) |
| Western Europe | 210051.70  (177764.63,  247275.08) | 187892.83  (150441.46,  236341.37) | 145.75  (123.34,  171.58) | 144.79  (115.93,  182.12) | -0.25(-0.56,0.06) |
| Western Sub-Saharan Africa | 5777.72  (4596.75,  7280.36) | 17562.78  (14108.28,  22050.57) | 8.07(6.42,  10.17) | 9.19(7.38,  11.53) | 0.43(0.41,  0.44) |
| SDI |  |  |  |  |  |
| High SDI | 408879.72  (346075.06,  489095.40) | 386135.57  (310562.08,  483223.76) | 117.84  (99.74,  140.96) | 109.31  (87.92,  136.80) | -0.46(-0.72,-0.20) |
| High-middle SDI | 113893.50  (93410.83,  140032.20) | 121414.54  (97321.36,  153522.34) | 25.17  (20.64,  30.94) | 27.58  (22.11,  34.87) | 0.61(0.27,  0.96) |
| Middle SDI | 76673.40  (60795.15,  96791.16) | 151067.43  (119487.16,  194250.15) | 10.19(8.08,12.86) | 16.29  (12.88,  20.94) | 1.82(1.57,  2.06) |
| Low-middle SDI | 81735.45  (65157.14,  104415.02) | 178219.44  (140023.74,  228725.61) | 18.03  (14.37,  23.03) | 22.21  (17.45,  28.50) | 0.87(0.75,  0.99) |
| Low SDI | 23172.54  (18292.93,  29550.56) | 61637.93  (48899.40,  79044.35) | 12.57(9.93,16.03) | 13.73  (10.89,  17.60) | 0.43(0.34,  0.52) |

**ASPR: Age-standardized prevalence rate，UI: Uncertainty interval，CI: Confidence interval，SDI: Socio-Demographic Index，EAPC: Estimated annual percentage change**
